# Past and current asbestos exposure and future mesothelioma risks in Britain: The Inhaled Particles Study (TIPS)

**DOI:** 10.1093/ije/dyx276

**Published:** 2018-03-09

**Authors:** Clare Gilham, Christine Rake, John Hodgson, Andrew Darnton, Garry Burdett, James Peto Wild, Michelle Newton, Andrew G Nicholson, Leslie Davidson, Mike Shires, Tom Treasure, Julian Peto, TIPS Collaboration, Andrew Duncan, Michael Dusmet, John G Edwards, Eric Lim, Richard Milton, Ian Morgan, P O’Keefe, Danielle Power, P B Rajesh, Sridhar Rathinam, Doris M Rassl, T Routledge, Michael Shackcloth, Anthony De Soyza

**Affiliations:** 1 London School of Hygiene and Tropical Medicine, London, UK; 2 Health and Safety Executive, Bootle, UK; 3 Health and Safety Laboratory, Buxton, UK; 4 Department of Cardiovascular Sciences, University of Leicester, UK; 5 Department of Histopathology, Royal Brompton and Harefield Hospitals NHS Foundation Trust, and National Heart and Lung Institute, Imperial College, London, UK; 6 Department of Cellular Pathology, Leeds Teaching Hospitals NHS Trust, Leeds, UK; 7 Leeds Institute of Cancer and Pathology, University of Leeds, UK; 8 Clinical Operational Research Unit, University College Hospital, London, UK

## Abstract

**Background:**

Occupational and environmental airborne asbestos concentrations are too low and variable for lifetime exposures to be estimated reliably, and building workers and occupants may suffer higher exposure when asbestos in older buildings is disturbed or removed. Mesothelioma risks from current asbestos exposures are therefore not known.

**Methods:**

We interviewed and measured asbestos levels in lung samples from 257 patients treated for pneumothorax and 262 with resected lung cancer, recruited in England and Wales. Average lung burdens in British birth cohorts from 1940 to 1992 were estimated for asbestos-exposed workers and the general population.

**Results:**

Regression analysis of British mesothelioma death rates and average lung burdens in birth cohorts born before 1965 suggests a lifetime mesothelioma risk of approximately 0.01% per fibre/mg of amphiboles in the lung. In those born since 1965, the average lung burden is ∼1 fibre/mg among those with no occupational exposure.

**Conclusions:**

The average lifetime mesothelioma risk caused by recent environmental asbestos exposure in Britain will be about 1 in 10 000. The risk is an order of magnitude higher in a subgroup of exposed workers and probably in occupants in the most contaminated buildings. Further data are needed to discover whether asbestos still present in buildings, particularly schools, is a persistent or decreasing hazard to workers who disturb it and to the general population, and whether environmental exposure occurs predominantly in childhood or after beginning work. Similar studies are needed in other countries to estimate continuing environmental and occupational mesothelioma hazards worldwide, including the contribution from chrysotile.

**Key Messages**

- Occupational and environmental mesothelioma risks from asbestos in older buildings are not known. Airborne concentrations are too low and variable for lifetime exposures to be estimated reliably, and mesothelioma rarely develops within 35 years of beginning asbestos exposure.
- British mesothelioma death rates are proportional to the population’s average amphibole asbestos lung burden (lifetime risk 0.01% per fibre/mg).
- Occupational and environmental risks can therefore be predicted from the distribution of asbestos lung burdens in people who began work since the 1980s, when asbestos was no longer used.
- The lifetime mesothelioma risk from environmental exposure among people born since 1965 will be ∼1 in 10 000, 10-fold less than in older people and almost 1000-fold less than in carpenters born in the 1940s. The risk is an order of magnitude higher in a subgroup of exposed workers.
- Further data are needed to discover whether asbestos in buildings, particularly schools, is a persisting or decreasing hazard.

## Introduction

Britain’s mesothelioma rate is the highest worldwide and is still rising above age 70.^1^ Former construction workers, particularly carpenters, plumbers and electricians, are the main high-risk group.^2^ Most mesotheliomas develop more than 35 years after first asbestos exposure, so almost all recent cases are due to exposure before 1980 when asbestos was widely used, and only three of the 2542 mesothelioma deaths in Britain in 2015 were born after 1975.^1^ Building workers may still suffer substantial exposure when asbestos in older buildings is disturbed or removed, and the general population are potentially exposed in such buildings. However, the resulting mesothelioma risks are not known, as current occupational and environmental airborne concentrations are too low and variable for lifetime exposures to be estimated reliably. The aims of The Inhaled Particle Study (TIPS) were to determine whether the linear relationship between mesothelioma risk and asbestos lung burden in individuals^3^ is also seen in national mesothelioma death rates and population average burdens, and hence to predict future occupational and environmental mesothelioma rates from the lung burdens of exposed workers and of the general population born since 1965 who started work after 1980, when use of asbestos had virtually ceased in the UK. Chrysotile (white asbestos) fibres are ignored in our analyses which are based on amphibole fibres, mainly amosite (brown asbestos) and crocidolite (blue asbestos). Chrysotile causes a much lower mesothelioma risk than the amphiboles,^4–6^ but its effect cannot be estimated from our data because its half-life in the lung is too short^3^ for lung burden to reflect lifetime exposure. Chrysotile constituted 88% of UK asbestos imports between 1955 and 1990 but only 2% of asbestos fibres in the lungs of men with mesothelioma or lung cancer, born 1940–64.^3^ Whatever its effect, therefore, the dose-response estimate based on all asbestos fibres in the lung would be virtually the same as our estimate for amphiboles.

## Materials and Methods

The study was approved by South Thames Multicentre Research Ethics Committee.

Lifetime occupational histories were obtained by telephone interview from resected lung cancer and mesothelioma patients in a national case-control study as previously described,^2^^,^^7^ and also from 1005 unselected pneumothorax patients (648 men, 357 women) born between 1918 and 1996, recruited from 13 hospital centres in England and Wales. All eligible pneumothorax patients (aged 18 or over, with retained lung samples obtained at operation within the past 10 years) identified in these centres were invited by the local clinician to take part in a telephone interview. Overall 42% replied agreeing to be interviewed, of whom 91% gave consent for their lung material to be analysed. The lung burden study was restricted to participants born in 1940 or later. Normal lung tissue for transmission electron microscopy (TEM) analysis was excised from residual stored material from 262 lung cancers resected in 1999–2010 and at subsequent postmortem from 133 pleural mesothelioma patients in a previous study,^3^ and from 271 pneumothorax patients surgically treated in 2002–10 (a random sample of 251 stratified by year of birth, sex and centre and 20 additional men born since 1965 who had worked in construction). Asbestos fibres longer than 5 µm were counted by transmission electron microscopy (TEM). The analytical detection limit (lung burden per counted fibre) was reduced from 10 to 3.3 f/mg (fibres per milligram of dry lung) for the 165 (90%) pneumothorax patients born since 1965 with sufficient material available. Job titles were assigned to Standard Occupational Classification 1990 (SOC 90) and grouped into categories of similar mesothelioma risk, as in our case-control study.^2^ Subjects were assigned to the highest risk job category they had worked in irrespective of duration. We classified those who had ever worked in any of the five categories with elevated mesothelioma odds ratios in our case-control study,^2^ as having occupational exposure (carpenters; plumbers, electricians and painters; other construction workers; other high-risk work; and medium risk). Those who worked in none of these jobs are referred to as environmentally exposed, which includes any exposures from buildings they worked in. The Health and Safety Executive provided cumulative mesothelioma mortality rates to age 50 years in England, Scotland and Wales for each birth cohort from 1940–44 to 1960–64.

### Statistical methods

**Figure 1:**
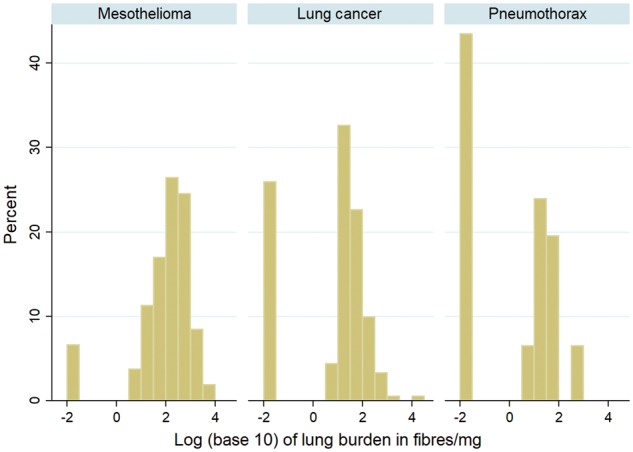
Approximately lognormal distribution of amphibole lung burdens in male mesothelioma, lung cancer and pneumothorax patients born 1940–64. Values < 5 f/mg are recoded as 0.01 f/mg, including 5/106 mesothelioma, 35/181 lung cancer and 14/46 pneumothorax samples in which no fibres were counted.

The distribution of lung burden is approximately lognormal (Figure 1) and fibre counts are modelled as Poisson. Mean population lung burdens in different subgroups in Tables 1 and 2 were therefore estimated by maximizing the Poisson-lognormal likelihood. Mean asbestos lung burdens in the general population born before 1965 were estimated using samples from lung cancer and pneumothorax cases. Asbestos increases lung cancer risk, so our analysis adjusts for this, using the previously estimated^3^ increase in lung cancer risk ratio (RR) with lung burden (0.00255 per f/mg) to estimate mean lung burden in the population from the observed levels in lung cancer patients. The linear relationship between cumulative mesothelioma mortality to age 50 and population mean lung burden was also estimated by maximum likelihood. To estimate the increase per f/mg in lifetime risk (defined as the actuarial probability of dying of mesothelioma by age 90), the slope was multiplied by 51.8, the ratio of projected lifetime risk to observed risk by age 50 in men. The statistical appendix gives further details. All tables, figures and analyses are restricted to amphibole fibres, except Table 3 and Figure 3 which also show chrysotile lung burdens.

**Table 1.** British mesothelioma mortality up to age 50 and population average amphibole lung burdens (f/mg) in the unselected sample by sex and year of birth

	Males						Females					
	Mortality to age 50		Mean lung burden (fibres/mg)		Fibres counted/ subjects		Mortality to age 50		Mean lung burden (fibres/mg)		Fibres counted/ subjects	
	Rate per million	No. of deaths	Mean^a^	95% CI	Lung cancer	Pneumothorax	Rate per million	No. of deaths	Mean^a^	95% CI	Lung cancer	Pneumothorax
1940–44	184	302	62.2^b^	(42.9, 91.8)	551/74	153/9	33	54	18.3	(11.2, 30.4)	87/26	0/1
1945–49	148	294	41.7	(30.5, 58.0)	394/66	54/13	29	58	13.3	(8.6, 21.2)	53/32	19/7
1950–54	99	180	30.8	(19.6, 49.0)	98/31	45/10	23	42	13.5	(7.1, 25.7)	19/15	11/6
1955–59	58	111	13.5	(5.8, 31.4)	25/7	6/7	22	44	10.8	(4.7, 25.2)	3/4	15/8
1960–64	35	63	10.9	(3.6, 32.0)	6/3	13/7	16	27	8.6	(3.6, 21.0)	8/3	8/7
1965–69			7.2	(2.3, 21.6)	1/1	9/8			1.2	(0.2, 4.4)		3/11
1970–74			3.3	(1.5, 7.0)		22/24			4.3	(1.7, 10.6)		14/11
1975–79			1.0	(0.3, 2.7)		6/21			1.2	(0.3, 3.3)		5/15
1980–84			3.2	(1.1, 9.1)		11/12			0.8	(0.2, 2.9)		3/12
1985–89			0.5	(0.1, 1.6)		3/21			1.0	(0.3, 2.7)		5/17
1990–92			0.0	(0.0, 2.4)		0/5			0.7	(0.03, 4.7)		1/5
Total					1075/182	322/137					170/80	84/100

a Lung burdens are adjusted for the effect of asbestos on lung cancer risk (see Statistical Methods). Respective unadjusted mean burdens in those born in 1940–44, 1945–49, 1950–54, 1955–59 and 1960–64 were 154.4, 52.0, 36.6, 14.8 and 11.7 f/mg in men and 20.2, 14.4, 14.6, 11.5 and 9.1 f/mg in women; respective unadjusted means based only on pneumothorax patients were 121.8, 17.8, 80.8, 1.6 and 15.0 f/mg in men and 0.0, 10.0, 16.2, 10.6 and 3.4 f/mg in women.

b Including a lung cancer with 22 000 fibre/mg.

**Table 2.** Average amphibole lung burden^a^ (fibres/mg) and 95% CI by occupation and year of birth in unselected lung cancer and pneumothorax patients and additional 20 construction workers with pneumothorax. (Number of fibres counted/number of subjects shown in parentheses.) The lower part shows the distribution of lung burdens by occupation and year of birth

	Occupational exposure							Environmental exposure only				
	Men						Women	Men	Women	Both sexes		
	Carpenter	Plumber, electrician or painter	Other construction worker	High risk	Medium risk	Any occupational exposure	Any occupational exposure			Observed	Predicted scenario A^b^	Predicted scenario B^c^
Mesothelioma OR v. population controls^d^	34.2	15.9	5.1	17.5	4.1		2.4	1.0 (ref)	1.0 (ref)			
Year of birth												
1940–54	154.3	87.6	29.7	59.8	49.2	56.4	13.5	19.6	15.2	16.9	18.5	19.6
	68.3–346.8	48.9–156.6	20.4–46.4	34.4–103.7	29.9–81.7	43.9–73.4	8.4–21.4	13.6–28.7	10.7–21.6	13.2–22.1		
	(217/12)	(264/25)	(204/48)	(297/31)	(207/41)	(1189/157)	(66/31)	(106/46)	(123/56)	(229/102)		
1955–64	78.0	15.6	2.1	0.0	11.7	22.7	8.9	5.9	9.4	7.9	6.3	7.4
	18.8–323.9	4.1–57.6	0.2–17.7		3.0–41.2	8.4–60.2	1.6–37.8	2.2–14.5	5.0–17.5	4.8–13.3		
	(20/2)	(11/4)	(2/2)	(0/1)	(7/4)	(40/13)	(3/4)	(10/11)	(31/18)	(41/29)		
1965–74	1.8	9.1	4.1		3.0	6.2	4.0	1.0	2.4	1.7	1.1	1.9
	0.2–9.1	3.7–21.7	1.5–10.7		1.0–7.9	3.0–12.8	1.0–13.7	0.3–3.1	1.0–5.4	0.9–3.4		
	(2/4)	(19/9)	(10/9)		(9/10)	(40/32)	(6/5)	(4/12)	(11/17)	(15/29)		
1975–84	1.7	9.1	1.4		0.5	2.9	2.5	1.2	0.9	1.0	1.1	1.0
	0.1–16.1	2.6–31.2	0.4–4.6		0.0–3.3	1.1–7.5	0.1–24.3	0.4–2.8	0.3–2.1	0.5–1.9		
	(1/2)	(9/4)	(4/9)		(1/6)	(15/21)	(1/2)	(7/19)	(7/25)	(14/44)		
1985–92		0.0	1.8		0.0	0.5		0.5	0.9	0.7	1.1	0.3
			0.1–16.9			0.0–4.7		0.1–1.4	0.3–2.2	0.3–1.4		
		(0/3)	(1/2)		(0/2)	(1/7)		(3/21)	(6/22)	(9/43)		
Lung fibre concentration f/mg												
Born 1940–64												
< 5	1	5	15	12	12	45	15	22	30	52		
5–24	2	9	17	7	17	52	14	21	29	50		
25–199	7	10	17	8	14	56	6	14	14	28		
≥ 200	4	5	1	5	2	17	0	0	1	1		
Born 1965–92												
< 5	6	8	16		16	46	6	51	60	111		
5–24	0	6	3		2	11	1	1	4	5		
25–60	0	2	1		0	3	0	0	0	0		

a Lung burden estimates are adjusted for the effect of asbestos on lung cancer risk, see Table 1 footnote a.

b Scenario A: annual accumulation of 0.1 f/mg per year from ages 5 to 16 from 1945 to the present, followed after age 16 by 1 f/mg per year until 1980 and zero since 1980.

c Scenario B: negligible exposure until age 16, followed after age 16 by 1 f/mg per year until 1980 and 0.1 f/mg per from 1980 until lung samples were obtained. For both scenarios, the calculation was based on individual years of birth and years of operation among those reporting no occupational exposure.

d ORs (odds ratios) from the case-control study.^2^

**Table 3.** Number and percentage of fibres counted by asbestos fibre type, year of birth, sex and occupation

		Number of fibres counted							% of fibres counted				Average lung burden f/mg^b^		
		Amphiboles						Chrysotile	Amphiboles			Chrysotile	Amphiboles		Chrysotile
	Fibre type^a^	am	cr	tr	an	ac	ua		am	cr	tr + an + ac + ua		am + cr	tr+ an + ac	
	*n* persons														
Men born since 1965															
Environmental only	52	6	2	4	1	1	0	5	31.6	10.5	31.6	26.3	0.5	0.3	0.3
Carpenter	6	3	0	0	0	0	0	0	100.0	0.0	0.0	0.0	1.6	0.0	0.0
Plumber, electrician, painter	16	26	1	0	0	1	0	0	92.9	3.6	3.6	0.0	6.8	0.9	0.0
Other construction workers	20	10	0	1	1	3	0	1	62.5	0.0	31.3	6.3	3.1	0.8	0.3
Medium-risk	18	7	0	1	1	1	0	2	58.3	0.0	25.0	16.7	1.3	0.6	0.4
Total	112	52	3	6	3	6	0	8	66.7	3.8	19.2	10.3	1.9	0.4	0.2
Women born since 1965															
Environmental only	64	14	2	3	4	2	0	5	46.7	6.7	30.0	16.7	0.8	0.5	0.3
Medium-risk	7	6	0	1	0	0	0	0	85.7	0.0	14.3	0.0	2.9	0.5	0.0
Total	71	20	2	4	4	2	0	5	54.1	5.4	27.0	13.5	1.0	0.5	0.2
Men born 1940–64															
Environmental only	57	62	14	12	21	5	3	11	48.4	10.9	32.0	8.6	11.4	4.8	1.7
High-risk	32	243	43	1	4	4	2	8	79.7	14.1	3.6	2.6	343.4	2.9	2.0
Carpenter	14	216	11	1	4	1	4	1	90.8	4.6	4.2	0.4	173.0	3.2	0.5
Plumber, electrician, painter	29	203	55	4	5	4	4	4	72.8	19.7	6.1	1.4	129.1	3.1	1.0
Other construction workers	50	170	17	1	8	5	5	13	77.6	7.8	8.7	5.9	30.0	3.1	2.5
Medium-risk	45	149	43	5	11	2	4	5	68.0	19.6	10.0	2.3	80.1	2.6	0.6
Total	227	1043	183	24	53	21	22	42	75.1	13.2	8.6	3.0	70.7	3.1	1.5
Women born 1940–64															
Environmental only	74	88	23	13	24	3	3	29	48.1	12.6	23.5	15.8	12.1	3.0	1.8
Medium-risk	35	46	8	2	12	0	1	7	60.5	10.5	19.7	9.2	12.2	3.2	1.3
Total	109	134	31	15	36	3	4	36	51.7	12.0	22.4	13.9	12.1	3.0	1.4

^a^Am, amosite; cr, crocidolite; tr, tremolite; an, anthophyllite; ac, actinolite; ua, untyped amphibole.

^b^Average lung burdens unadjusted for the effect of asbestos on lung cancer (see Statistical Methods).

## Results

**Figure 2:**
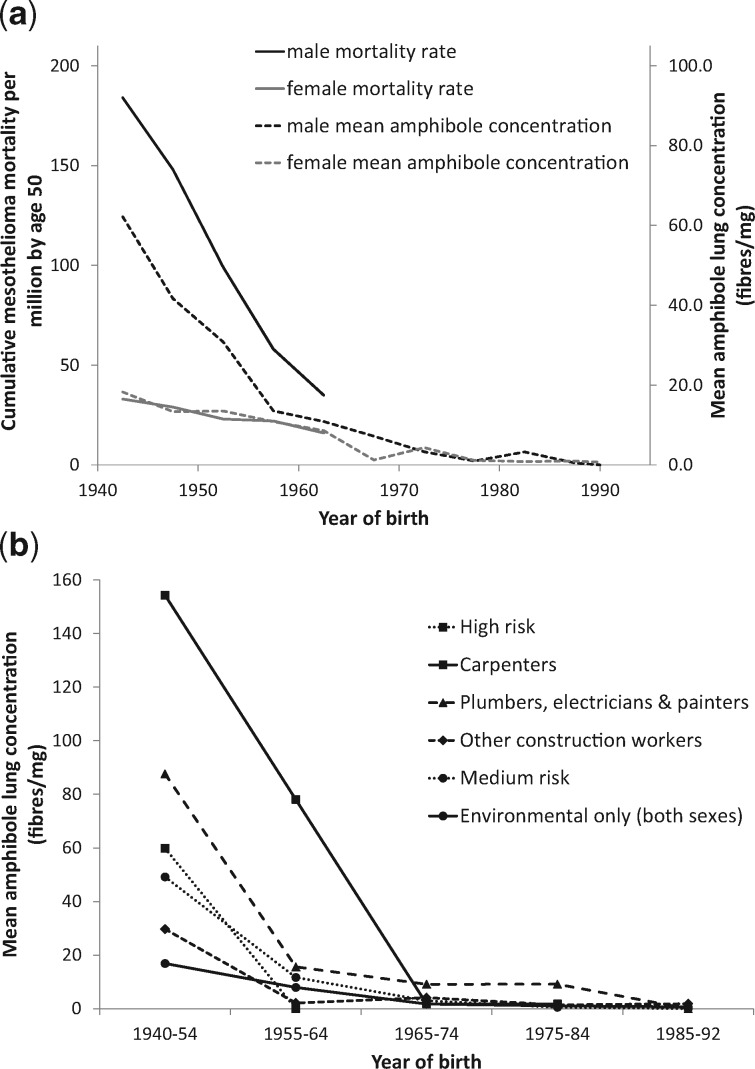
(a) National mesothelioma mortality and average amphibole asbestos lung burdens in Britain by year of birth (fibres/mg longer than 5 microns). Subjects born 1940–64 are predominantly resected lung cancer patients, whereas all but one of those born 1965–92 are pneumothorax patients. (b) Average amphibole asbestos lung burdens in occupationally exposed men by year of birth (fibres/mg longer than 5 microns). Data for environmental exposure only include both sexes.

In men, the average amphibole lung burden fell from 62 f/mg (born 1940–44) to 11 f/mg (born 1960–64) and mesothelioma risk per million to age 50 fell from 184 to 35 (Table 1, Figure 2a). In women, the average lung burden fell from 18 f/mg (born 1940–44) to 9 f/mg (born 1960–64) and their risk per million to age 50 fell from 33 to 16. The dose-specific mesothelioma risk to age 50 estimated from these data is 0.00032% per f/mg [95% confidence interval (CI) 0.00026%, 0.00040%)] for men and 0.00019% per f/mg (95% CI 0.00014%, 0.00024%) for women (*P* < 0.002). Average lung burdens unadjusted for asbestos-related lung cancer risk for those born 1940–64 are shown in Table 1 footnote a. (Only one lung cancer patient was born after 1964.) The adjustment has a material impact only for men born before 1955.

Table 2 and Figure 2b show lung burdens by year of birth and highest risk occupation. For environmental exposure (those who never worked in hazardous occupations), burdens were much lower and were similar in men and women. In those born 1940–64, the proportion with lung burdens exceeding 200 f/mg was 19% (14/75) among men who worked in the three highest risk categories (carpenters; plumbers, electricians and painters; other high-risk occupations), 2% (3/152) among other men and 1% (1/109) among women. None exceeded 60 f/mg in those born since 1965. Table 3 shows counts for each fibre type and unadjusted lung burdens for all amphiboles and chrysotile by year of birth, sex and occupation. In men, the overall distribution of counted fibres was 75% amosite, 13% crocidolite, 9% other amphiboles and 3% chrysotile, and in women 52% amosite, 11% crocidolite, 23% other amphiboles and 14% chrysotile. Fibre type differed between occupational groups, carpenters having the highest proportion of amosite (90.8%) and the lowest of crocidolite (0.4%). Chrysotile concentrations were uniformly low and showed no consistent relationship with occupation or gender.

**Figure 3:**
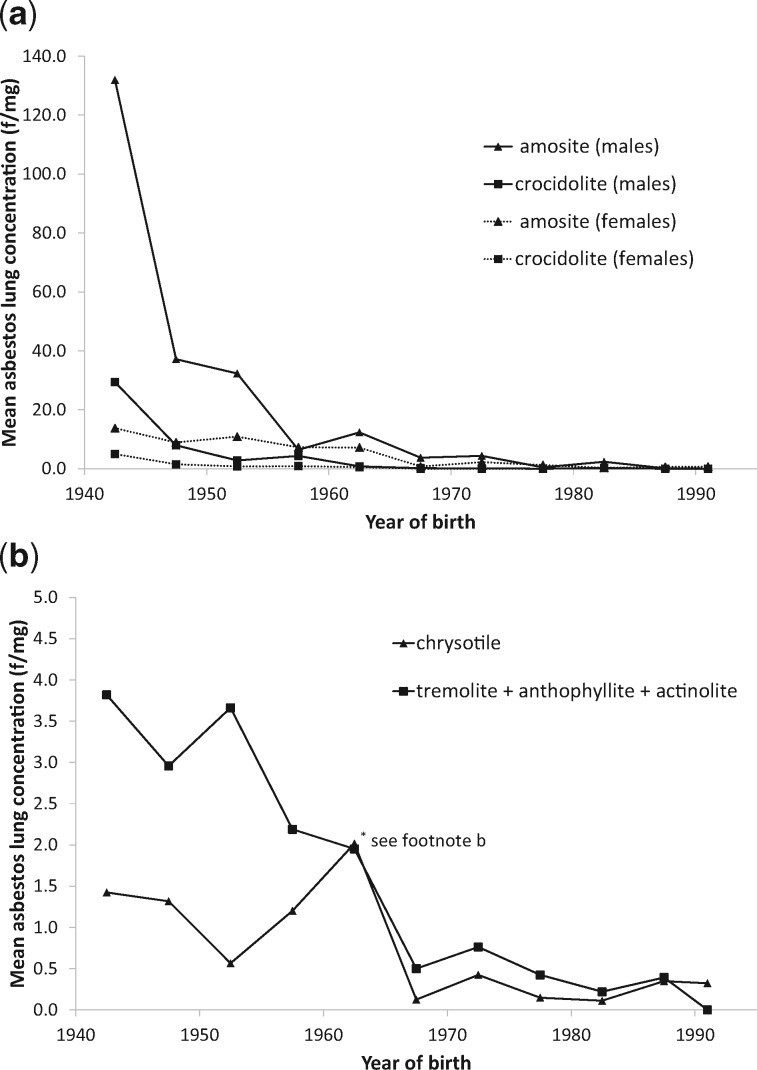
Average asbestos lung burdens^a^ in Britain by year of birth (fibres/mg longer than 5 microns). Upper graph: crocidolite and amosite by sex. Lower graph: other amphiboles and chrysotile (both sexes). ^a^Average lung burdens unadjusted for the effect of asbestos on lung cancer (see Statistical Methods). ^b^Excluding a chrysotile concentration of 72 f/mg based on 24 fibres in a woman who reported no asbestos exposure. Her inclusion increases the chrysotile average for those born 1960–64 from 2.0 to 26.0 f/mg.

People born in 1965–74 began work after 1980 when amosite materials were no longer being installed. Their average lung burden was as low in carpenters (1.8 f/mg) as in unexposed men and women (1.7 f/mg) but remained substantially higher among plumbers, painters and electricians (9.1 f/mg: Table 2, Figure 2b). Figure 3 shows that crocidolite burdens fell sharply in men born after 1950, about 5 years earlier than amosite.

## Discussion

### Trends in lung burden and dose-specific risk in those born before 1965

Average lung burdens in men born 1940–54 (Table 2) reflect the ranking of occupational and environmental relative risks seen in our case-control study^2^ (154 f/mg in carpenters, 88 f/mg in plumbers, electricians and decorators, 60 f/mg in other high-risk occupations (including shipbuilding and lagging), 49 f/mg in medium-risk (mainly factory) work and 30 f/mg in general construction). Lung burdens in those born 1940–54 with environmental exposure only were similar in men and women (average 17 f/mg). Occupationally exposed women had a similar level (14 f/mg, 95% CI 8, 31). Occupational and environmental lung burdens were substantially lower in those born 1955–64 but show a similar pattern.

Regression analysis of the parallel decline in mesothelioma mortality and average amphibole lung burden in male birth cohorts from 1940–44 to 1960–64 (Figure 2a, Table 1) gives a cumulative risk by age 50 in men of 0.00032% per f/mg. Multiplying by 51.8 (see Statistical Methods) gives a lifelong mortality of 0.017% per f/mg, close to the lifetime incidence of 0.020% per f/mg estimated from case-control analysis of lung burdens in male mesothelioma patients.^3^ However, the male data are dominated by a heavily exposed minority. The estimated increase in lung cancer RR from our case-control study (0.00255 per f/mg) is very imprecise,^3^ and adjusting for it substantially reduced the estimated average lung burdens of men born before 1955 (see Table 1 footnote a). Lung burdens in women are much lower and are hardly altered by the adjustment. Therefore we believe that the female estimate of the risk per f/mg (0.00019% by age 50, lifetime risk 0.010%) provides a more reliable indication of future mesothelioma rates in both sexes from recent exposure, which is predominantly environmental. This predicts a lifetime mesothelioma risk of the order of 1 in 10 000 at the average lung burden of ∼1 f/mg due to environmental exposure in men and women born since 1965 (Table 2).

### Asbestos exposure since 1980

By 1980, when those born in 1965 were starting work, traditional high-risk occupations such as lagging and shipbuilding had disappeared and carpenters no longer cut amosite board. The only occupational groups born since 1965 with substantially higher lung burdens than the general population are the 43% (6/14) of plumbers, electricians and decorators, 17% (3/18) of other construction workers and 14% (3/22) in medium-risk occupations in whom two or more amphibole fibres were counted in approximately 0.3 mg of lung tissue. The mean lung burden in these 12 cases (11 f/mg) implies a lifetime risk of ∼1 in 1000. Potentially remediable work practices seem likely to underlie this continuing occupational hazard. The distribution among the other 48 men and women in jobs classed as occupationally exposed in whom fewer than two fibres were counted, including the remaining eight plumbers, electricians and decorators, was 35 with no fibres and 13 with one fibre, similar to that among those with environmental exposure only. The reduction in the asbestos-exposed workforce and their declining lung burdens are reflected in the converging trends in male and female mesothelioma rates (Figure 2a). The majority of mesotheliomas in people born since 1965 will be caused by environmental exposure, presumably mainly in buildings. Numbers of amphibole fibres counted in 105 men and women born since 1965 with environmental exposure only (77 with none, 22 with one, four with two, one with three and one with four fibres) suggest fairly uniform environmental exposure across the UK, with a minority having higher (probably unsuspected) exposure. For example, these fibre counts are consistent with about 10% having a mean lung burden of ∼6 f/mg (lifetime risk ∼1 in 2000), with the remaining 90% having a lung burden an order of magnitude lower (∼0.6 f/mg; lifetime risk ∼1 in 20 000).

The steep decline in mean lung burden in men and women with environmental exposure only from 17 f/mg born 1940–54 to 1 f/mg born 1975–84 (*P* < 0.001) indicates that environmental as well as occupational exposure levels fell abruptly around 1980 when use of amphibole products had ended. This suggests that until the 1970s, most asbestos entered the environment during or soon after installation of new asbestos materials. Current environmental releases may also occur mainly during construction or demolition work on asbestos-containing buildings.^8^ (Our sample included no asbestos removal workers, but removal and demolition may contribute substantially to both occupational and environmental exposure.) However, airborne asbestos fibres released by weathering and everyday occupation of buildings may also be an important source of environmental exposure. Identifying asbestos in buildings that warrants containment or removal should continue to be a regulatory priority, but unnecessary asbestos removal may increase the number of fibres released to the environment.

The trend in average lung burden for men and women born before 1965 with only environmental exposure suggests an annual increment in eventual lung burden of ∼1 f/mg per year in adults until about 1980, when it fell sharply. The crucial question is whether environmental exposures, particularly in childhood, have remained fairly constant since 1980. In men and women born since 1965 with only environmental exposure, the average lung burden declines from 1.7 f/mg (95% CI 0.9, 3.4) born 1965–74, to 0.7 f/mg (95% CI 0.3, 1.4) born 1985–92 (*P* = 0.04), but the data are too sparse for the separate contributions of exposure in infancy, during school age and in adults to be estimated. Table 2 shows predicted lung burdens under two scenarios that are both consistent with these data but imply very different regulatory priorities: continuing exposure from age 5 to 16 with negligible environmental exposure after age 16 since 1980 (scenario A), and environmental exposure being negligible in childhood and beginning at age 16 (scenario B). Domestic exposure in infancy could be included without greatly altering these predicted lung burdens. The excess over these environmental levels in the average lung burdens of men with any occupational exposure increases for each year after age 16 by about 2 f/mg per year from 1955 to 1980, and after 1980 by about 0.1 f/mg per year in plumbers, electricians and painters, almost ceasing in other occupations (Table 2). UK amphibole imports up to 1980 show a similar pattern,^3^ changing little from 1960 to the late 1970s when amosite imports ended abruptly. Crocidolite use ended in 1970,^3^ and this is reflected in the earlier decline of crocidolite lung burdens in both sexes (Figure 3).

If asbestos levels have not fallen since the 1980s, our results suggest an average lung burden from current environmental amphibole exposure of about 1 f/mg by age 30. Lifetime mesothelioma risk is largely determined by asbestos exposure before age 30,^2^^,^^9^ and most of the amphibole fibres still present in the lungs of those born 1940–64, on whom our linear dose-response is based, were inhaled before age 30. However, the only direct evidence of recent environmental exposure is the average lung burden (0.7 f/mg) in 43 unexposed men and women born 1985–92 (Table 2), which is very imprecise and only includes fibres inhaled up to about age 19, the median age when their lung samples were taken. The 14 fibres counted in these 43 subjects comprised five amosite, one tremolite, one anthophyllite, two actinolite and five chrysotile.

### Study limitations

The consistency of the lung burden patterns in Table 2 with known occupational and environmental risks and national trends in mesothelioma mortality is reassuring. However, prediction of future risk from lung burdens in young adults may be affected by several factors. These include the proportion of environmental exposure that occurs in childhood, differences in amphibole fibre type and dimension between past occupational and current environmental exposure, and the opposite effects of fibre clearance and future accumulation on the lung burdens of those born since 1980 who were aged under 30 when samples were taken. Amosite has a particularly long half-life,^3^ but it is not known whether most fibres still present 20 years after inhalation remain in the lung forever, or whether carcinogenicity and clearance of tremolite, anthophyllite and actinolite are similar. Our main findings are unaffected by information bias, as the average lung burdens in Table 1 were based on the unselected sample irrespective of reported occupation. Any systematic differences between pneumothorax patients and the general population should have little effect on our prediction of future mesothelioma rates, if the dose-response in those born before 1965 and the lung burdens of younger people had both been based solely on pneumothorax patients. However, 78% of subjects born before 1965 were lung cancers from our previous study.^3^ The high cost of sample preparation and TEM precluded replacing them with pneumothorax patients, but differences between lung cancer and pneumothorax patients might lead to error in our prediction of future mesothelioma rates even if lung burdens in young pneumothorax patients were known precisely. Mean lung burdens in pneumothorax patients born before 1965 show no consistent difference from the overall estimates but vary irregularly across birth cohorts due to small numbers (Table 1 footnote a). The primary risk factor for both lung cancer and pneumothorax is smoking^10^^,^^11^ (among our participants 94% of lung cancers and 75% of pneumothorax patients had ever smoked), so marked differential bias related to the populations studied seems unlikely, but the lung sample was apical in almost all pneumothorax patients and from various sites in resected lung cancers. To avoid these uncertainties, future studies should use lung samples only from pneumothorax patients. This would also simplify the statistical analysis and might eliminate the difference between the results in men and women.

### Further studies and international comparisons

Lung burden studies on larger numbers of young people would determine whether environmental exposures have fallen since the 1980s and whether they occur predominantly in childhood or after beginning work. Analysis of larger amounts of tissue to increase sensitivity would identify individuals with higher levels that might be linked to specific buildings or other sources of environmental exposure. The mesothelioma risk from chrysotile is low^6^ but cannot be estimated from our results,^12^ and an international study of average TEM asbestos lung burdens is needed to show whether or not mesothelioma mortality in different birth cohorts can be explained by historical amphibole exposure even in countries where almost all asbestos was chrysotile. The risk per fibre for different amphiboles might also be estimated. Lower amphibole imports account for the much lower mesothelioma rate in the USA than in Britain and Australia,^2^^,^^3^ despite similar overall asbestos consumption per head. There is no consistent international correlation between overall asbestos consumption and mesothelioma risk, but crocidolite, amosite and chrysotile consumption were not recorded separately for most countries. Lin *et al*.^13^ reported a strong international correlation between the logarithm of recent mesothelioma mortality and historical asbestos consumption, which was predominantly chrysotile even in Britain. The exponential dose-response this would imply is interpreted as evidence of the mesothelioma risk from chrysotile,^14^ but the apparent correlation merely reflects two separate clusters of countries. There is little correlation either among the countries of North America, Australasia, Western Europe and Japan (the only outlier being Portugal) or in Eastern Europe, South America and the rest of Asia, where registered mesothelioma death rates and asbestos imports in the 1960s also varied widely but were much lower.^13^ This is confirmed in an updated analysis restricted to European countries.^15^ Replacement of chrysotile by safer substitutes is justified by the lung cancer and asbestosis risks, and the likelihood of some mesothelioma risk strengthens the case; but population-based data on amphibole lung burdens as well as total asbestos imports will be needed to identify any countries in which a large proportion of mesotheliomas were caused by chrysotile.

## Conclusion

The British mesothelioma death rate will decline from the current peak (0.75% of male deaths and 0.13% of female deaths in 2015) until about 2055, when those born before 1965 will be aged over 90.^16^ If the average lung burden by age 30 from environmental asbestos exposure is now ∼1 f/mg and remains at that level, there will be a continuing lifetime mesothelioma risk of the order of 1 in 10 000, averaged across the whole population. With projected population growth and ageing over the next 40 years, this would imply almost 100 mesotheliomas per year caused by asbestos, and there may be a similar number unrelated to asbestos.^17^ The risk is an order of magnitude higher in a subgroup of plumbers, electricians, decorators and presumably asbestos removal workers who do not take adequate precautions and probably in a minority of the general population with unusually high environmental exposure. Further samples from young people are needed to estimate current average lung burdens at each age more precisely. This would indicate whether the environmental hazard is declining and whether exposure is predominantly before or after school-leaving age. Our results suggest that a minority of the general population may have unusually high environmental exposure, but more sensitive fibre counting will be needed to confirm this. We are now recruiting further young pneumothorax cases, to identify those with high lung burdens so that their schools and homes can be studied.

## Funding

This work was supported by the British Lung Foundation (grant no. APG11-3), Health and Safety Executive and Cancer Research UK. A.G.N. was supported by the National Institute of Health Research Respiratory Disease Biomedical Research Unit at the Royal Brompton and Harefield NHS Foundation Trust and Imperial College London.

## Statistical Appendix

### Estimation of mean population lung burden adjusted for the effect of asbestos on lung cancer risk

Pharoah *et al.*^18^ considered a lognormal risk factor x where log(x) ∼ N(µ,σ^2^) in the general population and exposure-response is linear. They were modelling susceptibility to breast cancer, but as the log of asbestos lung burden is approximately normally distributed in the general population (see Figure 1) and we assume that the increase in lung cancer relative risk is proportional to lung burden,^3^ we can apply their results to our data. The distribution of x (i.e. lung burden) among cancers caused by asbestos will also be lognormal with log(x) ∼ N(µ+σ^2^, σ^2^). The arithmetic mean lung burden d in the general population equals exp(µ+σ^2^/2), so µ equals log(d)-σ^2^/2. Among the proportion p of lung cancers that are caused by asbestos log(x) ∼ N(µ+σ^2^,σ^2^), and the mean lung burden is d.exp(σ^2^). We assume that log(x) ∼ N(µ,σ^2^) among pneumothorax patients and among the proportion (1-p) of lung cancers that are not caused by asbestos. The lung cancer relative risk is 1+k.d. The estimate of k from our case-control analysis (k = 0.00255 per f/mg) was used.^3^

The lognormal variance σ^2^ may vary between birth cohorts, sexes and occupational groups, but is poorly determined in smaller individual cells. Accordingly, the cells in Tables 1 and 2 (and corresponding points in Figures 2a and b) were grouped, and the cells in each group modelled jointly with common group variance σ^2^ but different means µ(i), where i indexes the cells in the group. The groups were chosen such that the change in overall fit between fitting a different σ in each cell and fitting a common σ across cells was comfortably non-significant (*P* > 0.3). This resulted in three groups for Table 1 (men born 1940–44; men born later; and women) and five groups for Table 2 (other construction workers 1940–54; all other male occupations 1940–54; all male occupations combined 1940–92; environmental exposure, men and women combined; and all other cells).

In the ith cell in a group:

log(lung burden) ∼ N(µ(i),σ^2^) in the general population and in pneumothorax patients, d(i) = exp(µ(i)+σ^2^/2) = average lung burden in the general population and in pneumothorax patients, p(i) = k.d(i)/(1+k.d(i)) = proportion of lung cancers due to asbestos, and D(i) = p(i). exp(σ^2^).d(i) + (1-p(i)).d(i) = average lung burden in lung cancers.

For the jth individual in cell i, the true lung burden is X(i,j) fibre/mg and n(i,j) fibres are counted in w(i,j) mg of lung tissue, so n(i,j) ∼ P(n(i,j),[w(i,j).X(i,j)]) where P(n,λ) is the Poisson probability of observing n events with expected number λ.

Thus likelihood = ∏∏L(i,j), where for each lung cancer in cell i:

[Eqn 1] $$L\left(i,j\right)=\text{integral}\;\text{from}\;x=0\;\text{to}\;\text{infinity}\;\text{of}\;P\left(n\left(i,j\right),w\left(i,j\right).x\right).\left[p\left(i\right).g\left(x,µ\left(i\right)+\sigma^{2},\sigma^{2}\right)+\left(1-p\left(i\right)\right).g\left(x,µ\left(i\right),\sigma^{2}\right)\right].x^{-1}.d\left(x\right)$$

and for each pneumothorax patient in cell i:

[Eqn 2] $$L\left(i,j\right)=\text{integral}\;\text{from}\;x=0\;\text{to}\;\text{infinity}\;\text{of}\;P\left(n\left(i,j\right),\;w\left(i,j\right).x\right).g\left(x,µ\left(i\right),\sigma^{2}\right).x^{-1}.d\left(x\right)$$

where g(x,µ,σ^2^) is the lognormal function

(σ√2π)^−1^ exp-[(log(x)-µ)^2^/2σ^2^].

Replacing µ(i) by log(d(i))-σ^2^/2 in the likelihood gives maximum likelihood (ML) estimates of the population mean lung burden d(i) and its confidence interval in cell i. The ‘mle2’ function in the statistical package R was used to derive ML estimates, using package ‘poilog’ to provide the Poisson-lognormal likelihood. Confidence intervals were derived from the likelihood profile for each estimate. The likelihood shown in Equation 2 was used for lung cancers as well as pneumothorax patients, to calculate the unadjusted mean lung burdens in footnote a of Table 1, Figure 3 and Table 3. In Table 3, average lung burdens were calculated by fitting µ and σ separately in each cell.

### Estimating the relationship between national mesothelioma mortality and population mean lung burden

The slope b in the relationship M(i) = b.d(i) between average lung burden d(i) and cumulative mesothelioma risk by age 50 M(i) was estimated for each sex by maximizing the Poisson likelihood of the m(i) observed deaths in Britain in birth cohort i, given the population Pop(i) = m(i)/M(i), and the distribution of adjusted estimates of lung burden d(i) implied by the likelihood profiles for the five birth cohorts from 1940–44 to 1960–64 (Table 1).

For each sex, each of 5000 replicate estimates of b was derived by drawing values of d(i) for each birth cohort at random from the corresponding likelihood profile and fitting a Poisson regression with offset log(d(i).Pop(i)) to estimate the intercept log(b), so b is estimated by exp(intercept). The mean and 2.5% and 97.5% quantiles of these 5000 replicate estimates give (for each sex) the central estimate and 95% confidence limits for the risk coefficient b linking mesothelioma with average lung burden. The ratio of predicted lifetime risk (the actuarial probability of dying of mesothelioma by age 90) to the observed cumulative mortality to age 50 was estimated by simple age and birth cohort analysis of British male mesothelioma death rates from 1990 to 2009, assuming current British mortality rates for all other causes of death.

The statistical programming code is available on request.
